# Translating policy guidelines: a multiple case study of disease prevention in Sweden

**DOI:** 10.1186/s12913-025-13068-y

**Published:** 2025-07-07

**Authors:** Mattias Elg, Elin Wihlborg, Malin Wiger

**Affiliations:** 1 Division of Logistics and Quality Management, Department of Management and Engineering, Linköping University, 581 83 Linköping, Sweden; 2 Division of Policy Sciences, Department of Management and Engineering, Linköping University, 581 83 Linköping, Sweden

**Keywords:** Policy Translation, Implementation, Case Study, Public Healthcare System, Disease Prevention Methods, Multilevel-Multilogic framework

## Abstract

**Background:**

There is a growing need for new knowledge about healthcare policy implementation that is relevant to both researchers and practitioners. Many policy initiatives fail due to insufficient coordination between different system levels and conflicting agendas among various actors. This paper aims to propose and illustrate an analytical framework using a multilevel-multilogic framework. This framework helps reveal the combined challenges encountered when implementing policies within public healthcare systems.

**Methods:**

A multiple case study was conducted, focusing on the implementation of disease prevention guidelines in four Swedish healthcare regions. These regions were purposefully selected to represent diverse contexts and conditions that could influence policy translation. A total of 28 respondents across the four regions were interviewed, representing different system levels and institutional logics. The qualitative analysis identified connections between actors, settings, and policies, and explored how policy translation varied—from strong to weak, or even interrupted—as it moved from policy development to clinical practice.

**Results:**

We developed a theoretical and empirical understanding of policy translation processes, tracking how evidence-based national guidelines for disease prevention methods (DPMs) moved through regional administrative systems into clinical practice. The analysis focused on four main themes: the gradual translation and reinterpretation of policy objectives, the impact of shifting policy priorities, the facilitating role of technology in translation processes, and the ways policy became embedded into everyday clinical routines.

**Conclusions:**

Policy guidelines are implemented through a stepwise translation process, first being adopted and adapted within healthcare administrative systems via political and administrative activities, and then integrated into clinical practice. Within the multilevel-multilogic framework, each system level or logic has the potential to adapt, alter, delay, or even block the intended policy. Actions taken early in the translation process significantly affect the outcomes of subsequent stages.

**Supplementary Information:**

The online version contains supplementary material available at 10.1186/s12913-025-13068-y.

## Background

### Challenges in translating policy into practice

Recent research reviews display a growing interest in establishing new knowledge on evidence-based policy implementation that is relevant for both research and practice [1–3]. This concern is motivated by the difficulty of translating policy into practice [1, 4]. A central goal of policy research is to examine whether policies are implemented as conceived and to explain implementation success or failure [5]. This involves understanding how, why, and by whom policies are implemented and what outcomes they achieve [6, 7]. Implementation science is a dynamic field in healthcare research that plays a crucial role in closing the gap between evidence-based policy and its real-world application [8, 9]. By studying the link between evidence-based policies and their practical use, implementation science helps turn knowledge into actionable solutions [10]. Despite advances in this field, challenges remain due to the complexity of healthcare systems. The varied nature of healthcare systems makes it difficult to create one-size-fits-all strategies, requiring adaptations for each context [11, 12]. Some social science research even argues that the idea of straightforward implementation is too simple because it assumes change happens in a linear way [13, 14]. This type of approach to implementation science tends to result in lists of factors that do not connect to broader theories and concepts [10].

### The role of levels and logics in implementation

Previous research has identified several reasons why policy implementation is challenging, particularly highlighting the ongoing debate between top-down and bottom-up approaches within healthcare systems [1, 14]. More recent perspectives incorporate governance models and focus on the complex relationships among various actors through a multilogic approach [15, 16]. This approach recognizes that healthcare systems involve multiple groups of actors—such as politicians, administrators, and clinical professionals—each guided by their own distinct rationales, values, and priorities. For example, political actors may prioritize cost-effectiveness and population-level outcomes, administrators might focus on organizational efficiency and compliance, while medical professionals primarily value clinical autonomy and patient-centered care. These different rationales constitute the"multiple logics"that actors bring into policy implementation processes.

There is thus a potential in combining analyses of different system levels (macro, meso, micro) with multiple institutional logics to better understand challenges in policy implementation [17]. A central concept in this approach is that of *institutional settings*, which refer to the specific configurations within which actors operate. These settings are not purely political, administrative, or clinical in isolation, but are often hybrid constellations that blend elements from several domains. Such blended settings shape which actors are involved, how they interact, and to what extent they are able to influence the translation of policy across system levels. Consequently, policies that are effective in one institutional setting (such as a region with stable political leadership and aligned clinical support) might fail or encounter significant resistance in another (such as regions experiencing political instability or strong professional opposition) [18]. As policies move across different settings within healthcare systems, they often undergo shifts in form, meaning, and practical application—a process referred to as translation [17].

### Policy implementation as a translation process

We approach policy implementation as a translation process [19, 20], where evidence-based policies are developed and put into action through ongoing interactions among various actors in the healthcare system. In this view, implementation is a complex process that evolves over time through continuous collaboration and negotiation [21]. As policies are translated into action, different actors—such as policy-makers, healthcare providers, administrators, and patients—work together, each with their own interests and perspectives [22]. Thus, the process of policy translation can be understood as the iterative development of healthcare practices, organizational models, or service arrangements—where the original policy intentions are continuously adapted in response to changing circumstances, practical constraints, and the diverse roles, goals, and interpretations of the actors involved [23].

Actors play a crucial role in this process, as each one may serve as a translator—interpreting and reshaping ideas to align with the specific conditions, needs, and logics of their context [24–26]. During translation, policies may be modified or even rejected depending on the interests of the actors involved [24]. Every step in the process involves activities where actors contribute in different ways, all within various institutional settings that compete for attention and resources.

These institutional settings—such as political, administrative, and clinical environments—exist across all system levels (macro, meso, and micro) and shape the conditions under which actors operate [24]. In this sense, settings are not tied to a single level but interact with and influence how policies are interpreted and enacted at different levels of the system {23]. They shape actors’ capacity to adapt policies to their organizational realities and are therefore critical to successful implementation. For example, an intervention that appears promising at the macro (national policy) and meso levels may still be perceived as unfeasible or misaligned with priorities at the micro (clinical practice) level. Moreover, even within the same system level, different institutional logics can lead to diverging views—for instance, when policy officials and healthcare professionals interpret the same goals through different lenses [27]. These differing institutional logics set the rules and constraints that shape how actors interact in healthcare settings [28]. The logics are taken-for-granted rules that guide “field-level actors belief systems and related practices that predominate in an organizational field” [29].

We believe that three key logics are essential to understanding how healthcare guidelines are translated into practice. First, the political logic reflects the fact that public healthcare is governed by political decisions, which influence how care is provided, managed, and funded. Political norms, such as those based on the UN’s third sustainability goal to “ensure healthy lives and promote well-being for all at all ages,” guide overall healthcare practices and democratic decision-making [30]. Second, the administrative logic involves rational, bureaucratic control and emphasizes hierarchical decision-making. Managers and administrators operate within this logic, which both shapes and is shaped by other logics [22]. Third, the professional logic is based on medical expertise and professional autonomy. Healthcare professionals follow rules based on evidence and professional standards, relying on peer influence and professional accountability [31].

### Towards a multilevel-multilogic framework

**Fig. 1:**
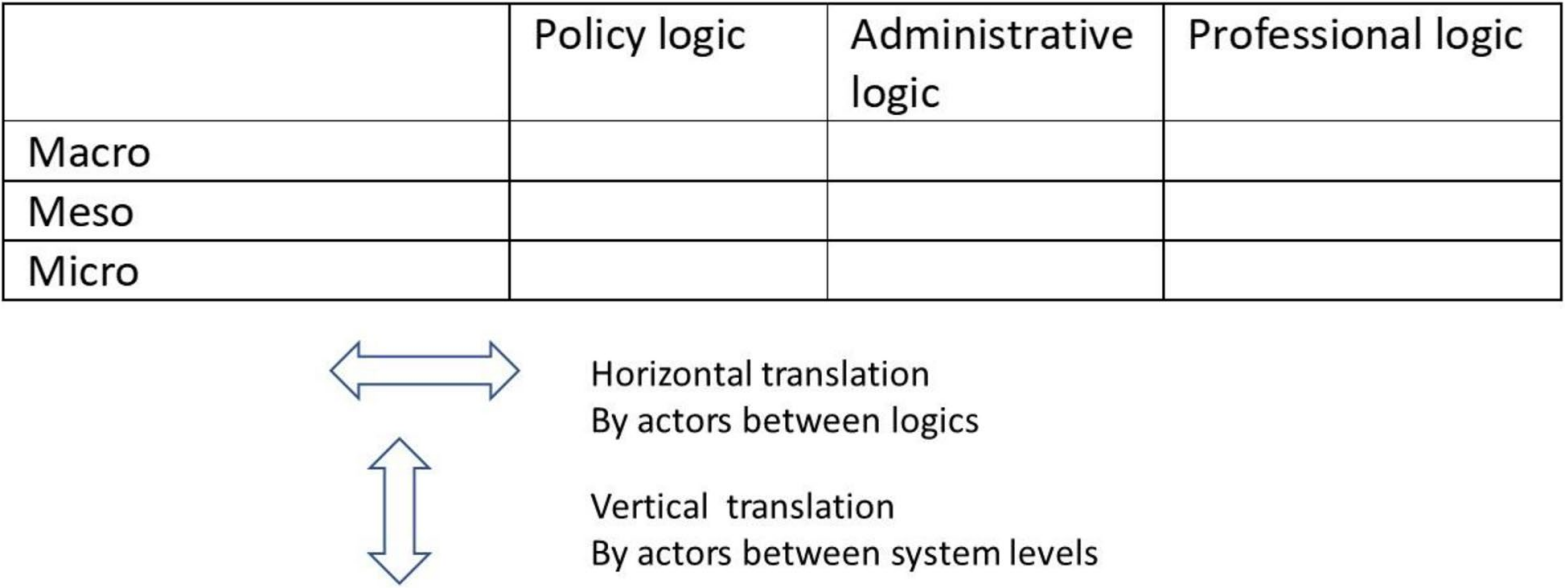
Multilevel-multilogic framework for the translation of policy guidelines

We assume that these three logics are present to varying degrees at different settings of the healthcare system – as shown in Fig. 1 below.

In this model, the macro level of the system consists of aspects that have legal and regulatory status at the highest system level. The meso level covers local health service and community factors. The micro level relates to day-to-day clinical practice. Policy translation unfolds through the interaction of these different levels, as actors contribute according to their respective capacities and resources [32]. This process involves not only vertical movement—where ideas and decisions are shaped across macro, meso, and micro levels—but also horizontal exchange between actors operating under different institutional logics.

Importantly, this study does not aim to offer empirically rigorous evidence of the effectiveness or causal mechanisms of policy implementation. Instead, our objective is primarily conceptual—to develop, illustrate, and refine the idea of a multilevel-multilogic framework. Recent translation research shows that ideas mutate as they travel between macro, meso and micro arenas under the pull of competing political, administrative and professional logics. Evidence ranges from a comprehensive review mapping three main strands of translation theory [33], a longitudinal hospital case revealing translators’ micro-tactics [34], an “ecology-of-roles” account of a leadership model’s journey [35], a micro-level study of sustainability work in hospitality [36], and a handbook chapter on the hybrid co-producer role in public services [37]—all illustrating the need for, and thus delimiting the novelty of, our multilevel-multilogic framework. A central idea shared by these studies—and by the present work—is that combining analyses of institutional system levels with the roles and logics of different actors helps pinpoint the junctures at which implementation may succeed or fail. The empirical examples we provide from a specific disease prevention program within Sweden's public healthcare system serve mainly as illustrative cases. Future research, including more rigorous empirical designs, is needed to validate and extend the framework’s applicability and utility across diverse contexts.

## Methods

We conducted a multiple case study of the implementation of the national initiative on disease prevention in four Swedish healthcare regions [38]. This study design allowed us to compare similarities, differences, and emerging patterns across cases, which improved the validity and generalizability of our findings. In this way, we could show where the policy implementation process varied or remained consistent across different regions. The policy we studied was based on national guidelines for disease prevention methods (DPMs) developed and adopted by the Swedish National Board of Health and Welfare (SNBHW). These guidelines were created by synthesizing evidence into evidence-based clinical policies in four areas: tobacco use, alcohol use, physical activity, and eating habits [39].

The Swedish healthcare system is publicly regulated and funded. Within the national legislation, control of the profession and soft-law knowledge-based governance, the regions have large autonomy regarding governance, management and regional adoption of national policies [40]. In international comparison, the Swedish healthcare system is highly decentralised [41]. The high degree of self-governance for the regions within a universalistic national healthcare system allows for a comparative study design within one state [42].

We selected regions across Sweden to reflect national diversity. Variation in size and geographical characteristics—including the inclusion of sparsely populated rural areas—were key selection criteria. We also considered the structures and processes used to manage and organize each region based on their self-governance opportunities Table 1.

**Table 1:** Central characteristics of the four regions

	South Border Region	Capital Region	East Sweden Region	Polar Region
Population^a^	1 378 000	2 377 000	465 000	250 000
Number of primary care units^b^	188	277	67	32
Number of full-time physicians in primary care per 1 000 inhabitants	0,8	0,6	0,7	0,9
Number of hospitals^b^	10	16	3	5

Sources: a) Statistics Sweden www.scb.se, b) National health care information 1177, www.1177.se

In our study, we interviewed 28 respondents from the four healthcare regions: East Sweden, Polar, South Border, and the Capital Region. The interviewees were identified in two stages: first, through nominations made by the Swedish National Board of Health and Welfare’s project lead for the national DPM guidelines, and second, via the main coordinators in each region, who pointed out additional key actors with formal responsibility for the implementation. In addition, all interviewees were purposively selected as key actors with responsibility for establishing the 2011 national DPM guidelines within their organisation (e.g., regional health directors, prevention strategists, clinical managers and policy-makers). Their diverse positions within their organisations provided valuable insights into the study, (see Additional file 1). The interviews were thematically oriented, following an interview protocol that was created specifically for this research (see Additional file 2). Each interview was recorded, transcribed, and anonymized to protect confidentiality.

Our research method tracked the development of guidelines from the national level through regional and local implementation, taking into account different institutional settings [24]. This approach allowed us to trace the motivations and reasons for actor involvement, as observed by independent researchers. A key advantage of this method is that it included a selection of actors involved with the guidelines, enabling us to follow their progression across various levels and logics.

**Fig. 2:**
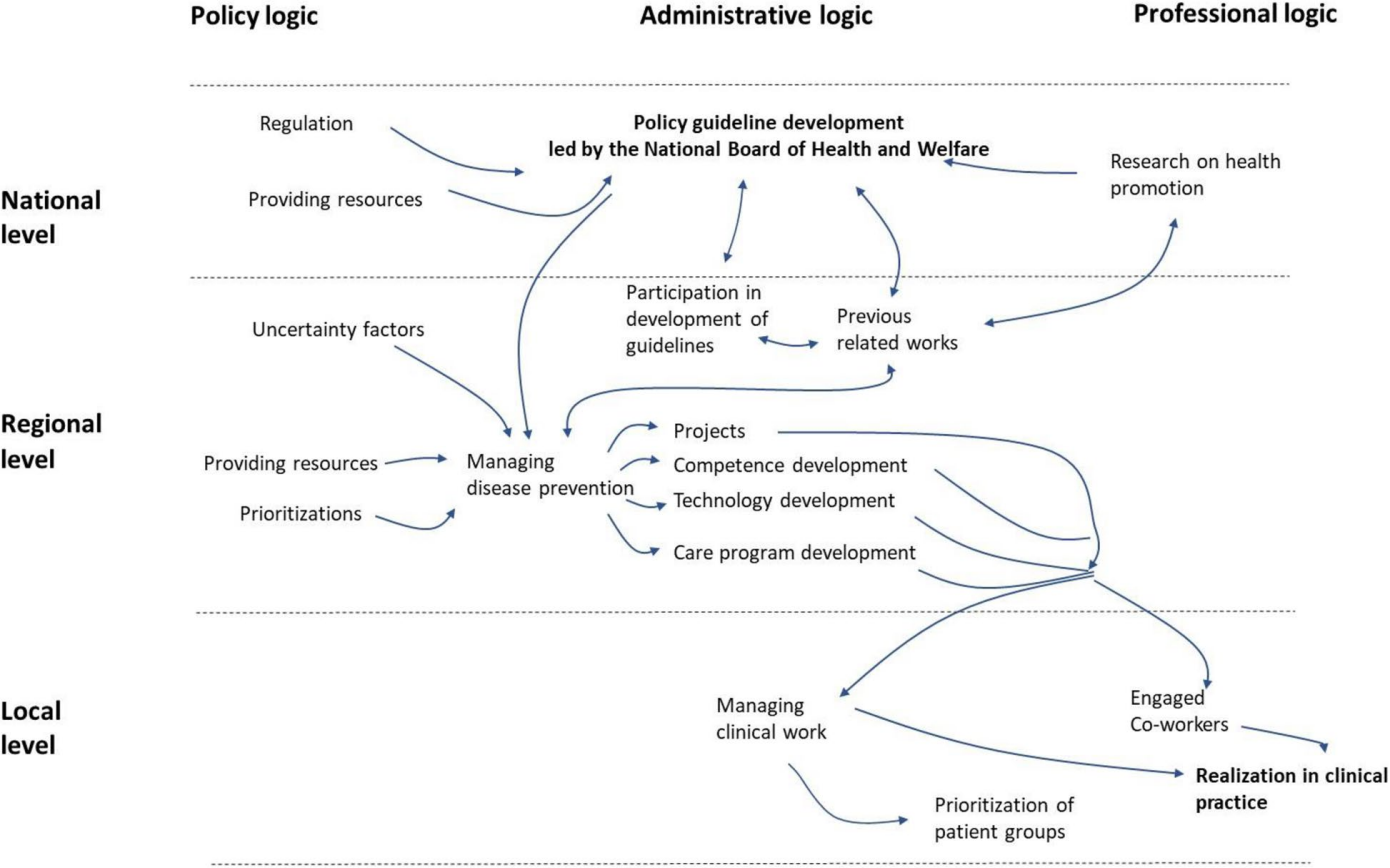
An overview of identified links in the translation of policy guidelines in the logic-level model

First, we identified each region’s approach to implementing disease prevention based on policy documents and interviews [43]. We then coded the interview transcripts using NVivo with an inductive approach. Next, we identified interconnected settings, actors, policies, and the chains of strong, weak, or broken translations from policy development to practice [44]. We labelled a translation as strong when the policy’s core intent was fully enacted: the planned activities were implemented, resources allocated, and responsibilities anchored in routine practice. A weak translation meant the policy was partly enacted—some activities or routines were adopted, but key elements (e.g., staffing, follow-up, or funding) were missing or sporadic. A broken translation referred to cases where the policy stalled entirely: no meaningful activities were launched or initial efforts were later abandoned. We then focused on the identification of common and general patterns, with the results being illustrated in a report in Swedish [43]. The research team collaborated to generate key thematic areas by combining their disciplinary perspectives to interpret the results and develop the multilevel-multilogic framework. This process involved synthesizing empirical findings into a broader analytical framework [44]. The study's results (especially Fig. 2) were validated in two workshops with the interviewees and other stakeholders in disease prevention in Sweden. All interview quotations were selected, translated and slightly linguistically edited by the authors.

## Results

In November 2011, the SNBHW approved national guidelines for disease prevention methods. These guidelines cover four areas: tobacco use, hazardous alcohol use, insufficient physical activity and unhealthy eating habits. Since then, the healthcare systems in Sweden, i.e. regions, have worked to implement these guidelines.

Figure 2 provides an overview of the translation process. It shows how the policy moves from national guidelines (macro level), through regional management (meso level), and finally to clinical practice (micro level), highlighting both strong and weak links in the process.

### Participation in guideline development: path dependency

Our analysis reveals that multiple logics come together during guideline development. At the national (macro) level, policies are initiated through regulation and resource allocation. Actors from both administrative and professional backgrounds collaborate on the guidelines, often drawing on previous experience from the regions. This prior work creates a clear pathway for implementation in each region.

It is evident that active actors played key roles throughout the healthcare system in the translation process. Rather than being chosen at random, there was a clear pattern—certain individuals were consistently tasked with initiating and integrating the guidelines into practice. For example, one senior manager in the South Border Region stated,

“[We have]… worked on networks and tackling development issues… and it remains as such… And then when the new guidelines came, it was the health promotion strategist – my predecessor – who stepped up to work on them.”

In the East Sweden Region, tobacco cessation work was considered particularly successful, providing a foundation for implementing the national guidelines. Their strategy in relation to the guidelines was based on “having carried out long and solid tobacco work in the East Sweden Region”, according to one of the respondents. The approach was perceived as structured and supportive, which simplified the translation of national guidelines into local practice. Thus, the guidelines were integrated into already ongoing activities and followed the established path set by previous tobacco cessation efforts.

This demonstrates that the main actors in the region adopted a proactive approach early on, preparing early for a smooth integration of the guidelines. Rather than being passive observers, they actively aligned the new guidelines with their existing activities, ensuring compatibility with regional structures and culture. By building on earlier implementation processes, the region facilitated networks for translating national guidelines into regional practice. Because these key actors shared the same administrative background—both nationally and regionally—the translation primarily occurred within the administrative logic, moving smoothly from national to regional levels. Also, clinical professionals were initially excluded, a fact later cited as justification for their limited involvement in guideline translation.

### What was translated? Shifting from individual disease prevention to broader public health promotion?

Most commonly, the guidelines were adapted to fit into the regions’ public health work. Regional actors often used concepts previously developed by public health centres. A policy administrator from the Polar Region said: “ we lean completely towards what they have been working with” indicating an alignment with existing public health activities. This broader health promotion perspective, rather than specific disease prevention, was embedded politically. A member of the regional council in the Polar Region explained:

“I believe that when the guidelines were introduced, they bolstered our existing work and reinforced our public health efforts. It made clear to us that our focus should be on prevention, not treatment. So, the question becomes, how do we create a comprehensive approach including preventive measures?”

Regional policy-makers felt free to decide how to proceed with the implementation. They appreciated the flexibility of the guidelines, which allowed them to integrate these guidelines into their ongoing public health and health promotion efforts. In East Sweden Region, for example, the guidelines were integrated into a health-screening program targeting healthy populations, with health calls offered to individuals at specific ages. However, this approach differed significantly from the guidelines'original intent, which focused on disease prevention at individual patient encounters. Regional actors did not clearly understand this distinction.

An analysis manager indicated that they faced substantial resistance from the medical profession, who felt burdened by the guidelines. Those who spearheaded the issues found themselves in a position where they had to justify their work. The manager also this:

“Perhaps the bulk of the resistance came from the medical professionals who felt they were also expected to implement these changes. The doctors critiqued the new guidelines, expressing concerns like “On top of all our existing duties, we’re expected to address this within a mere 15 minutes with the patient…”.

This forced regional health directors and public health managers to repeatedly defend the guidelines internally. Thus, adopting the guidelines required extensive collaboration among multiple key regional actors to align the recommendations with local professional practices. Despite the original focus on individual patient care, regional actors interpreted the guidelines as general public health initiatives. Consequently, the guidelines shifted towards broader health promotion, illustrating the flexibility and adaptability within the multilevel-multilogic framework.

### Designing activities at the regional level

While overseeing disease prevention at the regional level, administrators initiated a variety of activities. However, achieving consensus and involvement from all parties was challenging. In all regions, adapting the guidelines to professional practice was more complicated than expected. Certain groups gained influence, while others became less involved.

For example, the South Border Region required training in tobacco cessation counseling for personnel involved in disease prevention. This training provided the skills and formal recognition necessary to implement the guidelines effectively. However, issues arose when healthcare personnel without this training were tasked with counseling responsibilities. A regional health strategy manager explained:

“We observed many doctors could give general tobacco advice, but our program explicitly requires specialized tobacco cessation training. Yet, very few doctors have actually received this training.”

Interviewees characterized doctors’ lack of participation as passive resistance. By not engaging in required activities linked to the DPMs, doctors effectively allowed the guidelines to fade away without actively opposing them. Another interviewee noted that healthcare remains hierarchical, stating that getting doctors actively involved in disease prevention guidelines was nearly impossible.

This highlights a broader issue: compulsory administrative tasks intended to facilitate guideline translation may unintentionally provoke resistance among professionals.

### Policy reprioritisations and their impact

Despite passive resistance from some medical professionals, there was strong overall support for disease prevention. As a regional council member from the Capital Region remarked:

“We may have slightly different perceptions of what is the highest priority, but it is obvious that health promotion measures have strong support.”

The policy-makers provided symbolic legitimacy and resources for guideline implementation. East Sweden and Polar regions, with stable political leadership, prioritized disease prevention. In contrast, the South Border and Capital regions experienced political turbulence, negatively affecting guideline implementation. A medical adviser from the South Border Region described shifting political priorities:

“After the previous majority shift in the regional council, a lot of policy-makers changed… As a result, there are many new faces with different priorities.… Now the focus has shifted towards accessibility and care facilities and overcrowded emergency rooms…”

The reprioritisation in the South Border Region also led to considerable financial resources being deducted from disease prevention, which in turn undermined the adoption of the guidelines. When financial support for disease prevention dissolved, resources became embedded in the ordinary budgets – mainly within their already strained financial situation. The argument was that primary care was assigned more and more tasks while the total allocation of resources remained unchanged. The disease prevention activities became secondary to other initiatives. Despite the ambition of supporting the guideline efforts policy officials at the regional level failed to bridge the administrative logic to translate the guidelines into practice in the clinical healthcare work. A manager in the Capital Region pointed out this barrier:

“Numerous studies indicate that primary care’s proportion of resources has remained largely static, and it’s evident that primary care is increasingly burdened with follow-ups and checks for chronic illnesses. Despite these growing responsibilities, there are no additional resources. [When] having to handle larger tasks without the ability to change or maintain the same work methods, it’s clear that this aspect can get overlooked.”

Similarly, a medical doctor in the Capital Region questioned primary care’s role:

“Are we the ones who have the resources for this? Do we get the necessary financial resources needed to be able to carry out also this assignment in a good way?… There are numerous other entities well-equipped to handle primary preventive work, and it’s not clear to me that primary care should necessarily bear this responsibility.”

Thus, effective translation of guidelines required coordinated support across all regional and local levels, beyond mere policy prioritization.

### Implementing guidelines into clinical practice

A central goal of the guidelines was to change clinical practices to help patients adopt healthier lifestyles. However, translating guidelines into practical daily use proved challenging, partly due to uncertainties about their application. Healthcare strategists coordinated implementation, yet clinical professionals expressed concerns. One healthcare strategist from the South Border Region stated:

“I had significant reservations about the Swedish National Board of Health and Welfare indicators, questioning the necessity of precisely measuring the number of standard drinks a person consumes to initiate an intervention… Certainly, open-ended discussions are valuable, as patients might open up and admit, ‘I might be drinking a bit too much’.”

Medical professionals, used to nuanced patient interactions, felt guidelines overly simplified clinical assessments. A general practitioner from the Capital Region explained:

“I really have no problem with the legitimacy of these questions, nor do I think that many other people have, either. What was being questioned and discussed was our duty, so to speak, to address these issues in all contacts with patients, because it became a bit black or white. It was a [professional] group that was pushing this hard, and they were very specific that we should always use our precious time with patients to ask questions about living habits.”

Numerous policies are incorporated into daily clinical operations, and medical professionals’ express resistance towards their ability to integrate all these elements into their brief encounters with patients. Thus, different logics are hampering this translation, even if there is agreement with the evidence-based core values of guidelines.

Professionals faced difficulties incorporating guidelines into brief clinical visits alongside numerous other requirements. Therefore, embedding guidelines within regular organizational structures was crucial. Local leadership played a vital role in maintaining translation efforts. One healthcare strategist stressed:

“It should ideally be anchored in existing processes. And that is happening – there is a lot of process work everywhere.”

Effective local management was essential in clarifying how guidelines related to other concurrent initiatives. Additionally, technology supported guideline translation efforts.

### Technology supporting guideline implementation

Within our framework, technologies—such as coding systems and web-based templates—are viewed as material carriers of institutional logics. Typically designed at the meso level by administrative actors, they embody a rationale of standardisation and performance monitoring (administrative logic). Yet they must be enacted within the professional logic that governs everyday clinical work at the micro level. Because these artefacts are stable enough to preserve core policy intentions while still flexible enough for local adaptation, they operate as socio-material mediators that move and embed guidelines across system levels and logics throughout the implementation chain. Technology significantly facilitated guideline translation, helping clinical professionals integrate guidelines into daily work. Technological developments, primarily regional, included structured coding, reporting systems, web-based support, and workflow guidance.

Early guideline implementation involved coding activities to evaluate performance and manage knowledge. However, coding created confusion, as a primary care manager noted:

“We have to work with countless codes, action codes in the journal… Physical activity consists of four different codes, simple advice, as well as more developed advice and specialist advice … And then the prescription of physical activity. You have to know which of them to click. Did I give the patient a simple advice? You do not really know so you skip the registration.”

In East Sweden Region, technology advanced further through the Health Chart, a digital patient record integration that standardized documentation of lifestyle habits. An analysis manager emphasized its benefits:

“Previously, documentation was somewhat disorganised; questions about tobacco use were scattered across the many templates in the journal system, making it hard to extract relevant statistics. To address this, we developed a standardised template based on the guidelines. This template has a consistent structure and set of questions, enabling national comparison and making the work more transparent.”

Technology thus became a practical medium, effectively translating guidelines into usable tools for clinical professionals. However, several interviewees highlighted ongoing data analysis challenges, including difficulties aggregating quality data for preventive activities. A medical adviser from the South Border Region noted:

“it is very difficult, almost impossible, to pick data at the aggregate level. Unfortunately, they have not delivered the output data tools we have requested. We are trying to find out how to do it and create it ourselves here centrally. We have even been given some government funding to create a system for generating quality indicators at the primary care level.”

Technologies can translate meanings across the different levels and logics. The design of such technology is critical, since it must fit into both the logic and the ambitions of the sending and receiving contexts to help actors to associate their practice with the core meaning and values of the implementation programme. The selected disease prevention technologies vary among the regions, and due to the self-governance of the regions, there were no national guidelines on the technologies for translations.

## Discussion

Our core contribution is conceptual: we propose and illustrate a *multilevel-multilogic* framework that explains how policy guidelines travel from national intent to clinical routine. Drawing on translation theory from social science and policy studies [19, 20], we analysed Sweden’s disease-prevention guidelines to show how actors situated at the macro, meso and micro levels negotiate—and sometimes clash over—political, administrative and professional logics. We address this gap by proposing and demonstrating the value of a multilevel-multilogic framework. In successful cases, guidelines were first integrated into administrative healthcare systems at each setting and subsequently implemented in daily clinical practice through political and administrative actions. Similar approaches to process analysis can be found in policy sciences and organizational theory [20, 34].

Our findings extend the translation literature—and demonstrate what translation theory informed by organisation studies can add to policy-implementation research—in three ways. First, we show that regional administrators, not national policymakers or frontline clinicians, occupy the pivotal translator position. Where Wæraas and Nielsen mapped the types of translation processes that might occur [33], our multilevel-multilogic lens captures those processes in motion: the same regional officials who adapt national guidelines to local conditions also shape the micro-tactics that eventually reach clinical settings. This translation work was not coincidental. In each region, a core group of *hybrid professionals* [45]—with one foot in administrative systems and one in professional or policy networks—assumed the role of active mediators. These actors were not merely carriers of policy but strategic facilitators, using their contextual knowledge and institutional position to align national intent with local capacity. Their bridging function helped manage tensions between levels and logics, particularly where policy ambitions were ambiguous or politically sensitive.

Second, we show how early, path-setting decisions have lasting effects. The local tactics described by Waldorff and Madsen [34] are, in our study, made possible by key decisions taken early in the implementation chain. For example, appointing experienced prevention strategists, aligning new activities with long-standing public health initiatives, and leveraging existing networks helped anchor the guidelines within regional routines. These findings support the idea of *path dependency*, where existing institutional and organisational conditions shape what is seen as feasible or legitimate. In regions with stable leadership and policy continuity (such as East Sweden and Polar), this alignment facilitated a smoother translation. In contrast, where political support weakened (e.g., South Border), translation became more fragile and fragmented. Our study shows that translation is not just a response to guidelines, but often a continuation of ongoing agendas and commitments already embedded in the organisational system.

Third, we bring the material dimension to the forefront: technology emerged as a decisive enabler in the translation process. Workflow-compatible digital tools—such as structured templates, coding systems, and reporting platforms—supported the movement of guidelines from administrative intent to clinical routine. These tools helped standardise expectations while enabling local adaptation, and were especially powerful when they were integrated into existing documentation or decision-support systems. Similar to Elmholdt et al.’s “ecology of roles” [35], we show how the enactment of institutional logics often depends on such artefacts. Technology was not just a neutral enabler; it actively shaped what parts of the guideline were visible, traceable, and actionable. In some regions, technology served as a stabilising infrastructure, while in others it became a source of confusion or resistance—particularly when it conflicted with professional logic or added to perceived administrative burden.

Our study also extends the empirical scope of translation research by showing that similar dynamics recur even in highly regulated public health systems, as also suggested by Linneberg et al. [36] and Mortensen et al. [37]. Importantly, we demonstrate that while vertical coordination (from macro to micro) is essential, *horizontal coordination*—bridging competing logics at the same level—is often more difficult and less visible. For example, aligning administrative and professional logics at the clinical frontline required not just top-down mandates but practical, negotiated processes within local teams. Resistance among local clinicians was rarely vocal or ideological; instead, it took the form of passive non-adoption or quiet re-prioritisation in everyday clinical work.

Applying our multilevel-multilogic framework, we argue that successful guideline implementation depends on a complex interplay of institutional logics, organisational levels, material infrastructure, and strategically positioned actors. Smooth translation occurred when new guidelines resonated with existing practices and beliefs, and when implementation could draw on earlier investments, technologies, or networks. Where such alignment was absent, even well-designed policies struggled to gain traction. The framework helps uncover not only *whether* translation happens but also *how*, *by whom*, and *under what conditions*—adding depth to existing models of implementation.

In this way, translation theory informed by organisation studies offers more than conceptual nuance—it provides concrete analytical tools for identifying leverage points and barriers in policy implementation. It draws attention to the informal negotiations, socio-material structures, and historically embedded roles that shape whether a policy directive results in meaningful change. For researchers and practitioners alike, this framework enables both zooming out to map overarching translation pathways and zooming in to examine the friction points where policy either embeds or unravels.

Ultimately, by combining insights from translation theory and organisation studies, our multilevel-multilogic framework moves implementation research beyond simplified, linear models. It supports a more relational, dynamic, and context-sensitive understanding of how evidence-based policy is reinterpreted, redirected, or reinforced as it travels across complex systems like healthcare.

### Limitations

This study has several limitations. First, the sociocultural and political context of Swedish healthcare—characterized by specific norms, policies, governance models, and practices—may limit the transferability of our findings to other healthcare systems or national contexts. Although our analytical framework offers general insights, its applicability to other institutional settings needs further empirical validation through comparative research.

Second, we only interviewed 28 individuals across four healthcare regions, which constrains our ability to fully capture all nuances and complexities of the implementation processes. Consequently, important stakeholder perspectives, particularly those from clinical professionals who might have more critical or dissenting views, may not be fully represented. Future research should consider including larger and more diverse samples of stakeholders to better capture the full range of perspectives, resistance patterns, and implementation challenges encountered in clinical practice.

Third, our data collection was cross-sectional and relatively short in duration, preventing us from assessing long-term changes, adaptations, or the sustainability of the guideline implementation processes. Longitudinal research would be valuable to understand how policy guidelines evolve over time, how stakeholder engagement changes, and how sustainable implementation efforts are over extended periods.

Fourth, the design of our multiple case study was primarily exploratory and descriptive, which means it cannot fully establish causal relationships or evaluate the effectiveness of specific implementation strategies or interventions. Subsequent studies could adopt mixed-method designs, incorporating quantitative approaches, controlled trials, or longitudinal evaluations to provide stronger evidence on the causal mechanisms and effectiveness of policy translation strategies.

Fifth, as researchers with backgrounds partly outside the clinical health domain, we approached policy implementation from a broader policy science and organizational perspective, which may differ from traditions and conventions common within health research. This could have influenced both our analytical lens and interpretations, potentially leading to overly broad conclusions or insufficient attention to clinical-specific issues. Future studies might benefit from interdisciplinary collaborations that more explicitly integrate clinical expertise and health services research perspectives to deepen understanding and enhance applicability within healthcare contexts.

In summary, while the study provides valuable insights through the multilevel-multilogic framework, caution should be exercised in generalizing or overstating the conclusions. Further research is clearly needed to validate, refine, and expand the framework by incorporating patient and clinician perspectives, increasing the number and variety of cases studied, and employing longitudinal and mixed-method designs to strengthen empirical and theoretical contributions.

## Conclusion

Through this study we have proposed a comprehensive multilevel-multilogic framework to enhance understanding and effectiveness of policy implementation in healthcare, focusing on the critical roles of actors and technology in bridging various organisational levels and logics. This framework underscores the importance of translation processes through cross-level integration and overcoming challenges of policy translation over multi-logics to ensure its effective implementation across diverse healthcare settings.

## Supplementary Information

Additional file 1: Respondents, their organisation and role.

Additional file 2: interview protocol.

## Data Availability

The datasets used and analysed during the current study are available from the corresponding author. The study builds on a report published in Swedish and may be downloaded from the following link: http://urn.kb.se/resolve?urn=urn:nbn:se:liu:diva-128098

## Abbreviations

DPM: Disease prevention methods
SNBHW: Swedish National Board of Health and Welfare
