# Supplementary material for: Translating policy guidelines: a multiple case study of disease prevention in Sweden
Source: BMC Health Serv Res. 2025 Jul 7;25:938. doi: 10.1186/s12913-025-13068-y (PMC12235762; doi:10.1186/s12913-025-13068-y)
Supplement: Supplementary file 1 — Additional file 1: Respondents, their organisation and role. [file 12913_2025_13068_MOESM1_ESM.docx]

# Additional file 1: Respondents, their organization and role

### Interviews

| Region | Date  2015 | Function | Length of interview |
| --- | --- | --- | --- |
| East Sweden region | 01-oct | Manager, unit of health analysis | 1 h |
| East Sweden region | 14-oct | Clinical manager, primary care | 1 h |
| East Sweden region | 23-oct | Clinical manager, primary care | 1 h |
| East Sweden region | 09-nov | Process coordinator, health | 0,5 h |
| East Sweden region | 09-nov | Process coordinator, health | 1 h |
| East Sweden region | 10-nov | Politicians, chair of new development council | 0,5 h |
|  |  |  |  |
| Polar region | 06-oct | Development leader, health promotion | 2h |
| Polar region | 06-oct | Clinical manager, primary care | 1 h |
| Polar region | 06-oct | Politician, majority leader, Green Party | 1 h |
| Polar region | 21-oct | Organisational developer | 1 h |
| Polar region | 21-oct | Manager Unit for public procurement | 2 h |
| Polar region | 21-oct | Clinical professional, nurse | 1 h |
| Polar region | 22-oct | Organisational developer | 1 h |
|  |  |  |  |
| South border region | 12-sept | Politician, Green Party | 20 min. |
| South border region | 30-sept | Public health strategist | 40 min. |
| South border region | 29-oct | Manager health strategy | 1 h |
| South border region | 14-oct | Clinical manager, primary care | 1 h |
| South border region | 25-sept | Development manager | 1 h |
| South border region | 13-octt | Clinical manager, primary care | 1 h |
| South border region | 13-oct | Medical advisor, primary care | 1h |
| South border region | 14-oct | Clinical manager, primary care | 1 h |
| South border region | 14-oct | Health promotion strategist | 1 h |
| South border region | 20-oct | Medical advisor, primary care | 1 h |
|  |  |  |  |
| Capital region | 20-oct | Clinical manager, primary care | 1 h |
| Capital region | 21-oct | Politician, Liberal Party | 1 h |
| Capital region | 22-oct | Strategist, Unit for public procurement | 1 h |
| Capital region | 22-oct | Clinical manager, primary care | 1 h |
| Capital region | 19-oct | Medical doctor | 1 h |
| Capital region | 21-oct | Project leader disease prevention | 1 h |
| Capital region | 01-oct | Project leader | 2 h |
| Capital region | 3-nov | Medical doctor, clinical manager, primary care | 1 h |
