# Supplementary material for: Translating policy guidelines: a multiple case study of disease prevention in Sweden
Source: BMC Health Serv Res. 2025 Jul 7;25:938. doi: 10.1186/s12913-025-13068-y (PMC12235762; doi:10.1186/s12913-025-13068-y)
Supplement: Supplementary file 2 — Additional file 2: interview protocol. [file 12913_2025_13068_MOESM2_ESM.docx]

Interview protocol – disease prevention methods

## Presentation of the project

# Questions

### Introduction

Please, can you briefly tell us who you are? What role do you have in your healthcare organization?

What is the general opinion about disease prevention in your healthcare system?

### Organization of the work

Can you tell us about the work you are doing on disease prevention? (why, what areas are you focused on? What methods are used?)

Is there a standardized process or procedure for the work with disease prevention? Is it mandatory or just a recommendation?

How have you practically organized the work with disease prevention? Who is doing the job? When and how? What kinds of supporting technologies are used (e.g. checklists, part of embedded work processes, IT systems internally or directly towards patients)

### Resources and steering of the work

How is the work with disease prevention financed?

What types of resources do you have for supporting healthcare professionals in the disease prevention? (education, data/information support, decision support systems, financial resources, extra personnel?)

How do you steer the disease prevention methods embedded in regular healthcare steering models? (e.g. integrated in goal deployment, budget processes, balanced scorecard, formal contracts, pay for performance).

How do you follow up the results/processes of disease prevention? How is performance measurement used in various management contexts for decision making? Do you use other models for evaluation?

### Involvement

How are patients involved in disease prevention?

How are relatives involved in disease prevention?

### Influential role models

Which are your role models as to organizations being successful in the area of disease prevention?

### Summing up the important factors

What are the most important aspects that makes make disease prevention successful in your organization? (e.g. ask about professionals involvement in how to organize the work, how management is involved in the work, legitimacy among professionals?

What are the obstacles?

### The future

What are the most important development areas you are seeing in disease prevention?

Anything we have missed?
